# Estimation of the transpulmonary pressure from the central venous pressure in mechanically ventilated patients

**DOI:** 10.1007/s10877-024-01150-5

**Published:** 2024-03-21

**Authors:** Federico Franchi, Emanuele Detti, Alberto Fogagnolo, Savino Spadaro, Gabriele Cevenini, Gennaro Cataldo, Tommaso Addabbo, Cesare Biuzzi, Daniele Marianello, Carlo Alberto Volta, Fabio Silvio Taccone, Sabino Scolletta

**Affiliations:** 1 Department of Medicine, Surgery and Neurosciences, Anesthesia and Intensive Care Unit, University Hospital of Siena, Viale Bracci 10, Siena, 53100 Italy; 2 Intensive Care Unit, Department of Translational Medicine and for Romagna, Azienda Ospedaliera Universitaria di Ferrara, University of Ferrara, 44121 Ferrara, Italy; 3 Department of Medical Biotechnologies, University of Siena, 53100 Siena, Italy; 4 Department of Information Engineering and Mathematics, University of Siena, 53100 Siena, Italy; 5 Department of Intensive Care, Erasme Hospital, Université Libre de Bruxelles, Brussels, 1070 Belgium

**Keywords:** Pleural pressure, Esophageal pressure, Transpulmonary pressure, Central venous pressure, Mechanical ventilation, Respiratory failure, ARDS, Digital filtering analysis

## Abstract

Transpulmonary pressure (P_L_) calculation requires esophageal pressure (P_ES_) as a surrogate of pleural pressure (Ppl), but its calibration is a cumbersome technique. Central venous pressure (CVP) swings may reflect tidal variations in Ppl and could be used instead of P_ES_, but the interpretation of CVP waveforms could be difficult due to superposition of heartbeat-induced pressure changes. Thus, we developed a digital filter able to remove the cardiac noise to obtain a filtered CVP (f-CVP). The aim of the study was to evaluate the accuracy of CVP and filtered CVP swings (ΔCVP and Δf-CVP, respectively) in estimating esophageal respiratory swings (ΔP_ES_) and compare P_L_ calculated with CVP, f-CVP and P_ES;_ then we tested the diagnostic accuracy of the f-CVP method to identify unsafe high P_L_ levels, defined as P_L_>10 cmH_2_O. Twenty patients with acute respiratory failure (defined as PaO_2_/FiO_2_ ratio below 200 mmHg) treated with invasive mechanical ventilation and monitored with an esophageal balloon and central venous catheter were enrolled prospectively. For each patient a recording session at baseline was performed, repeated if a modification in ventilatory settings occurred. P_ES_, CVP and airway pressure during an end-inspiratory and -expiratory pause were simultaneously recorded; CVP, f-CVP and P_ES_ waveforms were analyzed off-line and used to calculate transpulmonary pressure (P_L_CVP, P_L_f-CVP, P_L_P_ES_, respectively). Δf-CVP correlated better than ΔCVP with ΔP_ES_ (*r* = 0.8, *p* = 0.001 vs. *r* = 0.08, *p* = 0.73), with a lower bias in Bland Altman analysis in favor of P_L_f-CVP (mean bias − 0.16, Limits of Agreement (LoA) -1.31, 0.98 cmH_2_O vs. mean bias − 0.79, LoA − 3.14, 1.55 cmH_2_O). Both P_L_f-CVP and P_L_CVP correlated well with P_L_P_ES_ (*r* = 0.98, *p* < 0.001 vs. *r* = 0.94, *p* < 0.001), again with a lower bias in Bland Altman analysis in favor of P_L_f-CVP (0.15, LoA − 0.95, 1.26 cmH_2_O vs. 0.80, LoA − 1.51, 3.12, cmH_2_O). P_L_f-CVP discriminated high P_L_ value with an area under the receiver operating characteristic curve 0.99 (standard deviation, SD, 0.02) (AUC difference = 0.01 [-0.024; 0.05], *p* = 0.48). In mechanically ventilated patients with acute respiratory failure, the digital filtered CVP estimated ΔP_ES_ and P_L_ obtained from digital filtered CVP represented a reliable value of standard P_L_ measured with the esophageal method and could identify patients with non-protective ventilation settings.

## Background

Mechanical ventilation (MV) is a life-saving treatment in patients with acute respiratory failure; however, MV can also contribute to additional lung damage, which has been named “ventilator-induced lung injury” (VILI) [1]. To minimize VILI, current guidelines recommend a “protective” ventilation strategy, using low tidal volume and a plateau pressure < 30 cmH_2_O, especially in patients suffering from acute respiratory distress syndrome (ARDS) [2]. Nevertheless, these ventilatory settings might not be appropriate in all patients on MV [3, 4]; indeed, setting of MV parameters cannot rely solely on airway pressures evaluation, because this is an imprecise index of the stress applied to the lung, which is best estimated by the transpulmonary pressure (P_L_) [5]. The correct assessment of P_L_ would request the measurement of pleural pressure (Ppl), which is not easy to obtain in the clinical setting [6]. Thus, esophageal pressure (P_ES_), measured through an esophageal balloon, has been used as a surrogate of Ppl [7] and P_L_ calculated as the difference between airway pressure (P_aw_) and P_ES_ [6].

Despite the solid physiological background of P_L_, a large observational study showed that P_ES_ is rarely monitored in clinical practice [1]; this is due to difficulties in placing the esophageal balloon, validating the signal and interpreting the data [8]. For such reasons, an estimation of P_L_ available without the need of placing an esophageal balloon would be attractive for clinicians. Due to its low elastance and close anatomical location, superior vena cava could be an alternative site of measure of pleural pressure: previous studies have reported that tidal excursions in central venous pressure (CVP) may reflect pleural swings (ΔPpl) [9], although the comparison between ΔCVP and the reference method, i.e. ΔP_ES_ showed a poor agreement [10, 11]. Due to this uncertainty, the idea of using respiratory swings in CVP as a surrogate of ΔPpl has not been further implemented, and, to the best of our knowledge, only one recent study has attempted to estimate transpulmonary pressure from the respiratory fluctuations of CVP [12].

Some of the pitfalls in the CVP wave analysis are related to the assessment of CVP tracings. Indeed, CVP signal, as well as P_ES_, is influenced by the changes in intrathoracic pressure [9] and shows ventilation-dependent oscillations, mainly in the low-frequency domain. Although, this waveform also presents distortions related to the high-frequency cardiac oscillations and altered shape from peaks and troughs. As such, we developed a digital signal processing technique able to remove from the CVP signal the cardiac components and to extract therefore the respiratory ones; this method could be a simple and effective alternative to estimate P_L_ from the CVP analysis (f-CVP).

The aim of the present study was to assess the validity of CVP and filtered CVP respiratory swings (ΔCVP and Δf-CVP) as an estimate of esophageal respiratory swings (ΔP_ES_) in a cohort of mechanically ventilated patients with acute respiratory failure. Then, we obtained P_L_ using ΔCVP and Δf-CVP and compared them to P_L_ calculated with the reference P_ES_ method. For this purpose, we used the transpulmonary pressure formula that represents the tidal lung stress, the effective distending pressure of the lungs when they are inflated with tidal volume [13, 14]. Secondary outcome was to test the diagnostic accuracy of the f-CVP method to identify patients at risk of VILI with high P_L_ levels.

## Materials and methods

### Study population

This was a retrospective analysis of prospectively collected data from patients admitted to a 7-bed Intensive Care Unit (ICU) at the University Hospital of Siena (Italy). The study was approved by the local Ethics Committee (Comitato Etico Regionale per la Sperimentazione Clinica della Regione Toscana; PPL2-14925) and registered on Clinicaltrials.gov (NCT04443491). Written informed consent was obtained from the patient’s next of kin before starting the study protocol.

Between May 2016 and March 2019, all patients with a diagnosis of acute hypoxemic respiratory failure (defined as PaO_2_/FiO_2_ ratio below 200) requiring invasive mechanical ventilation and equipped with both a central venous catheter (CVC) and an esophageal balloon were enrolled within 48 h from the starting of MV. The decision to insert the esophageal balloon was made by the attending physician and P_ES_ was used to set MV, by reducing tidal volume (V_T_) (if P_L_ was equal or above 10 cmH_2_O) [15], by increasing tidal volume in case of respiratory acidosis (if P_L_ was within safe values), by adjusting PEEP level during a decremental PEEP trial test or to maintain an end-expiratory P_L_ equal or above 0 cmH_2_O or a combination of the above.

The exclusion criteria were: patients < 18 years; arrhythmias; presence of pulmonary air leakage (i.e. chest trauma and/or chest tube with active aspiration); pregnancy. Patients with arrhythmias were excluded due to difficult readings of the CVP trace. Chest tube with negative aspiration could impair homogeneous transmission of pleural pressure across the intrathoracic space, so those cases were excluded. Pregnant patients were excluded in according to the local Ethics Committee policy.

### Setting

During the measurements, patients were in the semi recumbent position. P_ES_ was measured using a double-balloon, graduated feeding catheter (NutriVent®, Mirandola, Modena, Italy) [16] connected to a pressure transducer filled with air; the esophageal balloon was initially positioned in the stomach to check for the presence of positive deflection; then, it was withdrawn until it reached the lower third of the esophagus when cardiac artifacts appeared on the esophageal pressure trace. To check the correct position of the esophageal balloon, external manual compressions on the rib cage were applied during an expiratory pause and simultaneous positive deflections of airway and esophageal pressures compared (i.e. “positive pressure occlusion test”) [6]. Briefly, an occlusion maneuver was performed at end-expiration; changes in P_ES_ (ΔP_ES_) and P_aw_ (ΔP_aw_) during gentle chest compressions were simultaneously recorded, and the test suggested correct positioning if ΔP_ES_/ΔP_aw_ ratio ranged from 0.9 to 1.1. Also, correct positioning was confirmed on chest X-ray, with the direct visualization of two radiopaque markers, one above and one below the diaphragm. The balloon was inflated at a volume of 4 ml according to the recommendations of the manufacturer [16] and the balloon inflation was checked before every measurement to ensure it contained the adequate amount of air.

A triple-lumen central venous catheter (CVC; Certofix Trio V720 Braun, Melsungen Germany) was placed, as routinely protocol in our department, using ultrasound and electrocardiogram (ECG)-guided technique, through the right jugular vein with the tip located in the superior vena cava upstream to the right atrium [17]. Correct positioning of the CVC was also confirmed on chest X-ray. Measure of CVP was obtained from the distal port of a fluid-filled CVC connected to an electronic pressure transducer connected to the bedside multiparametric monitor. The transducer was zeroed at the phlebostatic point at 5 cm below the sternal angle (thus at this level patients do not have to be supine for the measurements) [18]. CVP values were taken at the base of the “c” wave at the end-inspiratory and end-expiratory pauses [19].

Continuous ECG tracing, P_ES_ and CVP curves were displayed on the bedside multi-parametric monitor (Intellivue MP60, Philips), while simultaneously P_ES_ and CVP were acquired as “rough” signals by a dedicated software developed by the authors (LabVIEW, National Instruments Corporation, Assago-Milano, Italy) before entering in the ICU monitor [20]. All traces were acquired according to the default bedside monitor sampling frequencies (500 Hz for the ECG traces, 125 Hz for the others).

### Study protocol

All patients were deeply sedated (i.e. Richmond Agitation and Sedation Scale, RASS of -4 or -5 [21]), paralyzed and ventilated with a square flow waveform with tidal volume, respiratory rate and PEEP set by the attending physician, while the oxygen fraction was selected to keep a SpO_2_ between 94 and 96%. At baseline (T1), P_ES_ and CVP curves were simultaneously recorded for 5 min; if ventilator settings were changed, all measurements were repeated within one hour after such modification (T2). Each measure was taken after reaching an equilibrium (i.e. at least 15 min after the last change in ventilator setting) [22]. In all patients, an expiratory and inspiratory pause of 3–5 s were performed to achieve static respiratory mechanics. We recorded plateau pressure (P_plat_), total PEEP (PEEP_tot_) and intrinsic PEEP values. The driving pressure was calculated as P_plat_ minus PEEP_tot_ [23]. All these data were recorded as routine practice and therefore analyzed off-line.

### Off-line analysis

From a spectral analysis perspective, CVP signal is characterized by low frequency (respiratory-dependent) as well as high frequency (cardiac-dependent) components [24]. Therefore, we used a dedicated low pass digital filter to remove the high frequency components to obtain a f-CVP. Technical details regarding the low-pass filter design have been described in recent works [19]. Using a linear filtering approach, we assumed that CVP is the sum of three pressure components related to the heart activity (P_H_), the breath activity (P_B_) and other noisy components (N):

1 $${\rm{CVP}}\left( {\rm{t}} \right)\, = \,{{\rm{P}}_{\rm{H}}}\,\left( {\rm{t}} \right)\, + \,{{\rm{P}}_{\rm{B}}}\left( {\rm{t}} \right)\, + \,{\rm{N}}\left( {\rm{t}} \right)\, + \,{{\rm{P}}_0},$$

where P_0_ is a biasing pressure offset (the CVP mean value), whereas the remaining signals have zero-mean.

In the linear filtering approach, it is hypothesized that the pressures P_H_ and P_B_ are signals with separable high-frequency and low-frequency spectral components, i.e., it was assumed that for a certain frequency f0.

2 $${{\bf{P}}_{\bf{B}}}\left( {\rm{f}} \right)\, \approx \,0\,{\rm{if}}\,{\rm{ f}}\, > \,{\rm{f}}0,$$

3 $${{\bf{P}}_{\bf{H}}}\left( {\rm{f}} \right)\, \approx \,0\,{\rm{if}}\,{\rm{f}}\, < \,{\rm{f}}0,$$

where **P**_**B**_ and **P**_**H**_ are the Fourier transforms of P_B_ and P_H_, respectively. Following the above assumption, a theoretical linear low-pass filter with unity gain **H**_**LP**_ exists such that, neglecting the noisy term N, a reliable estimation **P´**_**B**_ of the spectrum **P**_**B**_ can be obtained as.

4 $${{\bf{P}}_{\bf{B}}}\left( f \right){\rm{ }} \approx {\bf{P}}_{\bf{B}}^\prime \left( {\rm{f}} \right)\, = \,{\bf{CVP}}\left( {\rm{f}} \right){{\bf{H}}_{{\bf{LP}}}}\left( {\rm{f}} \right)\,-\,{\bf{\delta }}\left( {\rm{f}} \right)\,{{\rm{P}}_0}$$

**Fig. 1:**
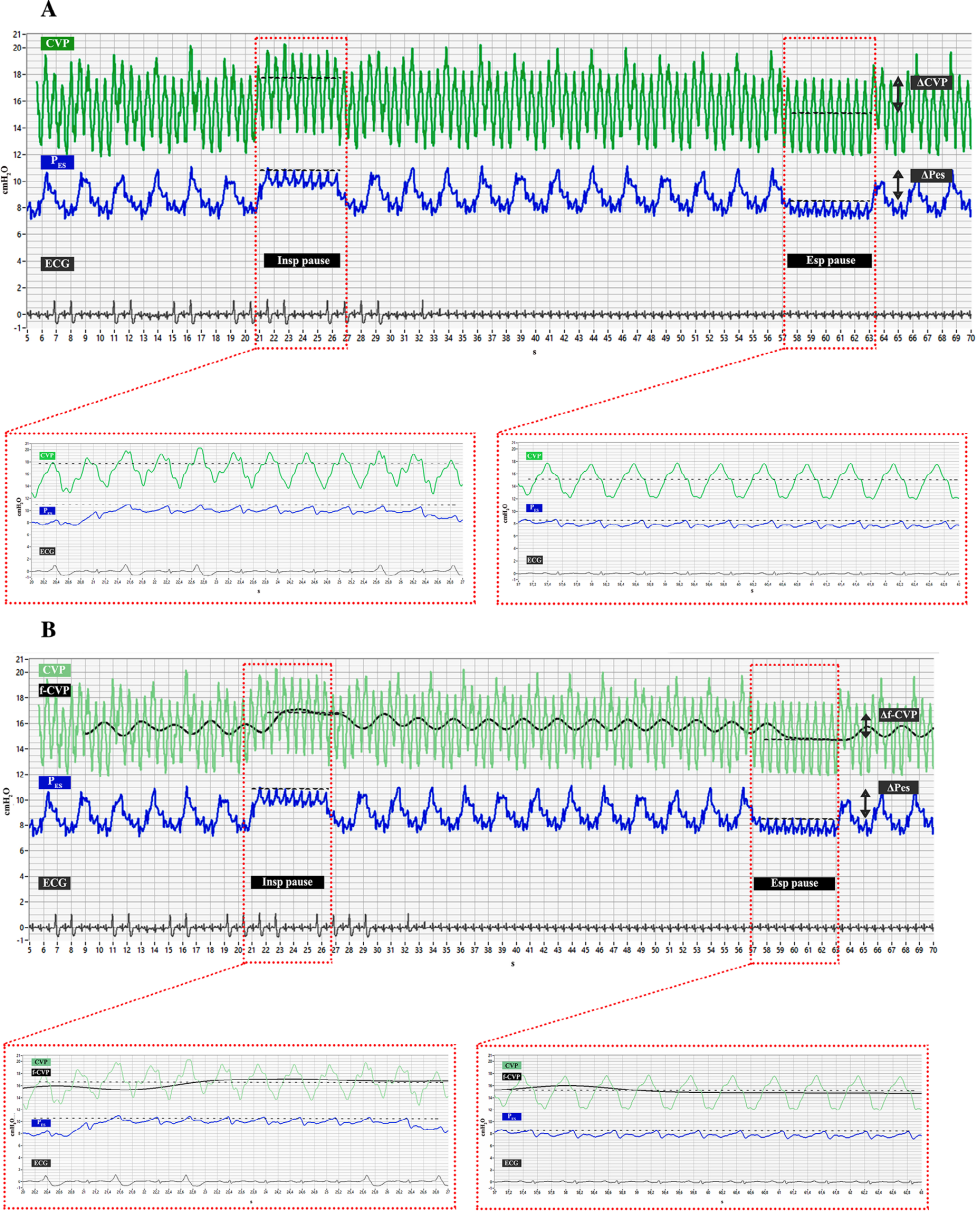
**A** showed the CVP curve (in green) and P_ES_ curve (in blue). ΔCVP and ΔP_ES_ were calculated as differences between values obtained during end-inspiratory and end-expiratory pauses. For each pause, CVP values were identified at the base of the “c” wave (the dotted horizontal line in CVP wave), while for P_ES_ positive peak values were considered (the dotted horizontal line in Pes wave). The double headed arrows in the CVP and P_ES_ curves identified the magnitude of ΔCVP and ΔP_ES_, respectively. In the red boxes the inspiratory and expiratory pauses were enlarged to appreciate the marker location. X axis, time as seconds (s); y axis, amplitude in cmH_2_O. **B** displayed the same curves in Fig. 1A with the addition of f-CVP curve (in black). Δf-CVP was calculated as differences between values obtained during end-inspiratory and end-expiratory pauses. For each pause, f-CVP values were identified at the plateau level (the dotted horizontal line in f-CVP wave). The double headed arrow in the f-CVP curve identified the magnitude of Δf-CVP. In the red boxes the inspiratory and expiratory pauses were enlarged to appreciate the marker location. X axis, time as seconds (s); y axis, amplitude in cmH_2_O

The values of f-CVP obtained during end-inspiratory and end-expiratory pauses were reported in the database and used to calculate transpulmonary pressure. Figure 1A and B shows examples of curves analysis with the proposed method: it could be noted that a temporal delay exists in the filtered curve due to the processing of the signal, however, for the purpose of the calculation of P_L_, this delay is negligible.

During the same respiratory pauses, we recorded end-inspiratory and end-expiratory values of P_ES_. These waveforms have cardiogenic oscillations: we measured the values of pressure at the peak of these oscillations for calculation. Filtered CVP, instead, appeared as a “clean” signal, without the typical waves, so we just selected the numeric values displayed during the respiratory pauses. Both P_ES_ and f-CVP waveforms were analyzed offline with LabVIEW software, as shown in Fig. 1.

The transpulmonary driving pressure was calculated as [13, 14]:

$$\eqalign{{{\rm{P}}_{\rm{L}}}{{\rm{P}}_{{\rm{ES}}}}{\mkern 1mu} = & {\mkern 1mu} \left( {{\rm{Pplat}}{\mkern 1mu} - {\mkern 1mu} {\rm{end} \ {inspiratory}}{\mkern 1mu} {{\rm{P}}_{{\rm{ES}}}}} \right){\mkern 1mu} \cr & - {\mkern 1mu} \left( {{\rm{total}}{\mkern 1mu} {\rm{PEEP}}{\mkern 1mu} - {\mkern 1mu} {\rm{end} \ {expiratory}}{\mkern 1mu} {{\rm{P}}_{{\rm{ES}}}}} \right) \cr}$$

The same formula was applied for CVP and filtered CVP obtaining P_L_CVP and P_L_f-CVP, respectively.

### Statistical analysis

Normal distribution of data was tested by the Shapiro–Wilk Normality Test. Data are reported as mean ± standard deviation or median [interquartile range] as appropriate. The paired t-test was used to compare the variables.

The association between ΔCVP and Δf-CVP with ΔP_ES_ and then between transpulmonary pressure values (calculated with P_ES_, CVP and f-CVP) were expressed by correlation coefficient (*r*). The agreement between ΔCVP and Δf-CVP with ΔP_ES_ and between P_L_ obtained with the reference P_ES_ method and with CVP and f-CVP was assessed using the Bland-Altman analysis corrected for repeated measures when appropriate. Limits of agreement (LoA) (as 2 times standard deviation, SD, of the bias) were computed.

The ability of f-CVP derived P_L_ to follow variations or trends in P_ES_ derived P_L_ after an adjustment in ventilator setting was assessed analyzing the correlation between the changes (∆) in P_L_ calculated by subtracting the first baseline from the second measurement (T2–T1), obtaining ∆P_L_f-CVP and ∆P_L_P_ES,_ respectively [25, 26]. We analyzed the direction of change between the pre-post differences to assess the percentage of concordance between the two methods. Two-tailed statistical hypothesis testing was performed with *p*-values ≤ 0.05 considered statistically significant.

The diagnostic accuracy of the f-CVP method to identify patients at risk of VILI was displayed as the area under the Receiver Operating Characteristic (ROC) curve (AUC). For this purpose, a threshold of 10 cmH_2_O of transpulmonary driving pressure was considered [15]. DeLong test was used to compare the AUCs of ROC [27].

As previously reported [11], compliance of the superior vena cava could influence the transmission of pleural pressure swings to the point that the ΔCVP relationship to ΔPpl decreased at higher values of CVP [10] and ΔCVP may underestimate ΔP_ES_. Thus, we tested the effect of CVP value on the correlation and agreement between the respiratory swings and the transpulmonary pressures by dichotomizing patients into two groups based on the median end-expiratory CVP value (low vs. high CVP level).

Statistical analysis was performed using MedCalc software (MedCalc Software bv, Ostend, Belgium; https://www.medcalc.org; 2016) and GraphPad PRISM version 6.0 (San Diego, CA, USA).

## Results

### Study population

During the study period, 163 patients had a diagnosis of acute hypoxemic respiratory failure requiring invasive mechanical ventilation and an esophageal balloon was placed in 46 (28%) of them. Due to technical issues on the data acquisition of the system software, the recordings of 22 patients, performed between October 2016 to December 2017, were lost. Thus, 24 patients were eligible for this study, but four were eventually excluded because of cardiac arrhythmia during the recording (*n* = 1) or poor quality of the waveform signals (*n* = 3). The remaining 20 patients represented the study cohort; demographic and clinical characteristics of these are shown in Tables 1 and 2; ARDS was diagnosed in seven patients, two with moderate and five with severe forms [28]. Rescue therapies were necessary in two patients (one required prone position and inhaled nitric oxide and another needed venous-venous extracorporeal membrane oxygenation); ICU mortality was 25% (*n* = 5). Ventilatory setting, as well as lung mechanics, determined before and after variation of the ventilator strategy, are shown in Table 3.

**Table 1:** Patients’ characteristics at ICU admission

Variable	Patients *n* = 20
Age, years	52 [46–63]
BMI (kg/m^2^)	27.5 [24.1–30.6]
Sex (M/F)	14/6
SAPS II score	39 [32–54]
SOFA score	8 [6–10]
*Comorbidities*	
COPD, n (%)	4 (20)
Hypertension, n (%)	9 (45)
Diabetes, n (%)	3 (15)
Cardiac disease, n (%)	3 (15)
*Reason for ICU admission*	
Sepsis or septic shock, n (%)	3 (15)
Pneumonia, n (%)	6 (30)
Trauma, n (%)	4 (20)
Neurological, n (%)	7 (35)
*ICU outcomes*	
Need for vasopressor, n (%)	15 (75)
Days of MV	19 [9–29]
Days of ICU	19 [9–33]
ICU mortality, n (%)	5 (25)
Need for tracheostomy, n (%)	12 (60)

Data are expressed as median [interquartile range, IQR] or number (percentage)

BMI, body mass index; SAPS, Simplified Acute Physiology Score; SOFA, sequential organ failure assessment score; COPD, Chronic obstructive pulmonary disease; ICU, Intensive care unit; MV = mechanical ventilation

**Table 2:** Blood gas analysis at study inclusion

Variable	Patients *n* = 20
pH	7.38 [7.30–7.42]
PaO_2_, mmHg	70 [59–78]
PaCO_2_, mmHg PaO_2_/FiO_2_, mmHg	46 [40–54] 117 [70–160]
HCO^3−^, mmol/l	25.2 [23.2–27.3]
BE, mmol/l	0 [-2; 5]
Lactate, mmol/l	1.7 [1.3; 2.7]

Data are expressed as median [interquartile range, IQR] and range

BE, base excess

**Table 3:** Ventilatory settings and lung mechanics

Variable	First assessment *n* = 20	Second assessment *n* = 16
*Ventilatory setting*		
Tidal volume, ml	460 [430–480]	480 [430–530]
Respiratory rate, breath/min	24 [18–26]	24 [18–27]
PEEP, cmH_2_O	11 [8–12]	10 [9–12]
*Lung mechanics*		
Plateau pressure, cmH_2_O Driving pressure, cmH_2_O	22.5 [19.5–25.3] 11.5 [8.5–13.3]	23.5 [17.8–26.0] 10.5 [8.8–12.3]
Respiratory system compliance, ml/cmH_2_O	44.8 [34.0–55.8]	45.5 [36.0–56.5]
Intrinsic PEEP, cmH_2_O	1 [0–1]	1[1–1]
P_ES_ inspiratory, cmH_2_O	15.3 [12.6–18.9]	15.6 [13.1–17.8]
P_ES_ expiratory, cmH_2_O CVP inspiratory, cmH_2_O CVP expiratory, cmH_2_O f-CVP inspiratory, cmH_2_O f-CVP expiratory, cmH_2_O ΔP_ES,_ cmH_2_O ΔCVP, cmH_2_O Δf-CVP, cmH_2_O P_L_P_ES_, cmH_2_O P_L_CVP, cmH_2_O	12.3 [10.6–15.2] 23.3 [15.0-27.3] 22.0 [12.8–26.1] 18.8 [12.3–21.7] 16.1 [9.8–19.7] 2.5 [2.0-3.2] 2.0 [1-1-2.3] 2.5 [2.1–2.8] 8.6 [5.9–9.8] 9.5 [6.2–11.8]	12.6 [10.8–15.0] 20.9 [15.3–27.4] 19.4 [12.2–24.5] 17.9 [12.2–20.6] 15.8 [10.0-18.4] 2.7 [2.0-3.3] 1.8 [1.2-3.0] 2.7 [1.8-3.0] 8.3 [5.8–10.4] 8.6 [6.7–10.2]
P_L_f-CVP, cmH_2_O	8.8 [6.0-10.2]	8.3 [6.0-10.2]

Data are expressed as median [interquartile range, IQR] or number (percentage)

PEEP, positive end expiratory pressure; P_ES_, esophageal pressure; CVP, central venous pressure; f-CVP, filtered central venous pressure; ΔP_ES_, ΔCVP and Δf-CVP, differences between inspiratory and expiratory values of P_ES_, CVP and f-CVP, respectively; P_L_P_ES_, P_L_CVP and P_L_f-CVP, transpulmonary pressure calculated from P_ES_, CVP and f-CVP, respectively

### Baseline measurements

**Fig. 2:**
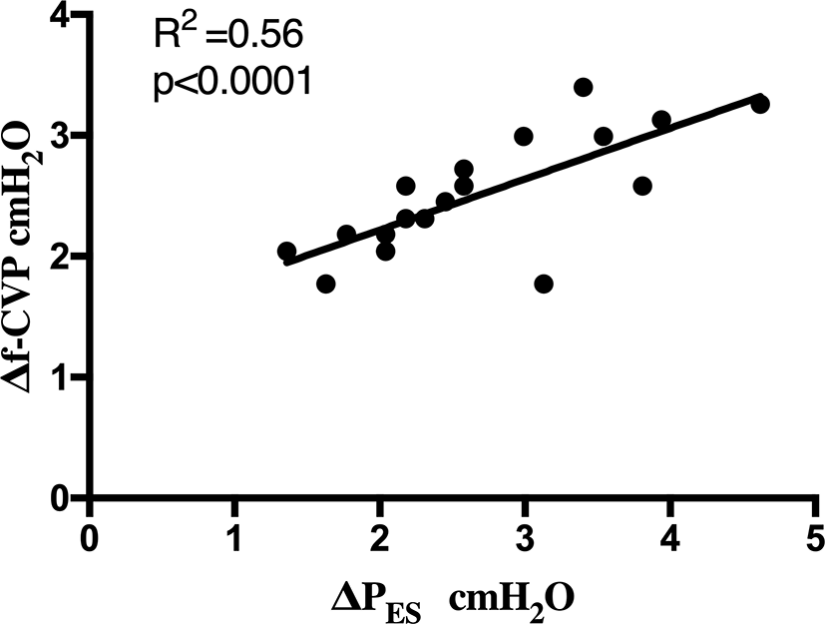
Correlation between Δf-CVP and ΔP_ES_. Δf-CVP, respiratory changes in filtered central venous pressure; ΔP_ES_, respiratory changes in esophageal pressure. Solid line, linear regression line

**Fig. 3:**
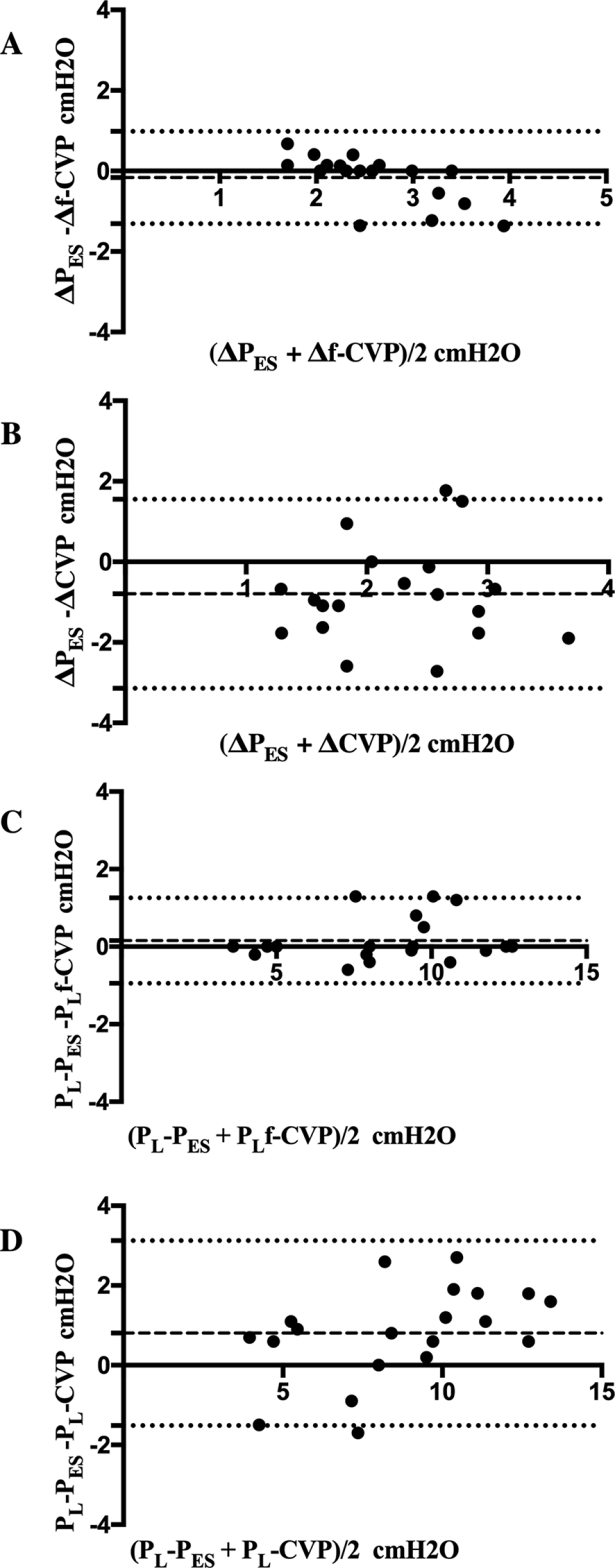
Bland-Altman analysis for the agreement between Δf-CVP and ΔP_Es_, and between ΔCVP and ΔP_ES_, box A and B, respectively. Bland-Altman analysis for the agreement between P_L_f-CVP and P_L_P_ES_, and between P_L_CVP and P_L_P_ES_, box C and D, respectively. Broken lines, bias; dotted lines, ± 1.96 SD of the bias. ΔCVP, respiratory changes in central venous pressure; Δf-CVP, respiratory changes in filtered central venous pressure; ΔP_ES_, respiratory changes in esophageal pressure; P_L_f-CVP, transpulmonary pressure obtained using f-CVP; P_L_P_ES_, transpulmonary pressure obtained using esophageal pressure; P_L_CVP, transpulmonary pressure obtained using CVP

Median values of P_ES_, CVP, f-CVP and relative respiratory tidal swings and transpulmonary driving pressures obtained are reported in Table 3. Δf-CVP (Fig. 2) correlated better than ΔCVP with ΔP_ES_ (*r* = 0.75, *p* = 0.001 vs. *r* = 0.08, *p* = 0.73 respectively). Also, the agreement with ΔP_ES_ was better for Δf-CVP than ΔCVP as revealed by the Bland-Altman analysis (mean bias − 0.16, LoA − 1.31, 0.98 cmH_2_O vs. mean bias − 0.79, LoA − 3.14, 1.55 cmH_2_O), as shown in Fig. 3A and B.

**Fig. 4:**
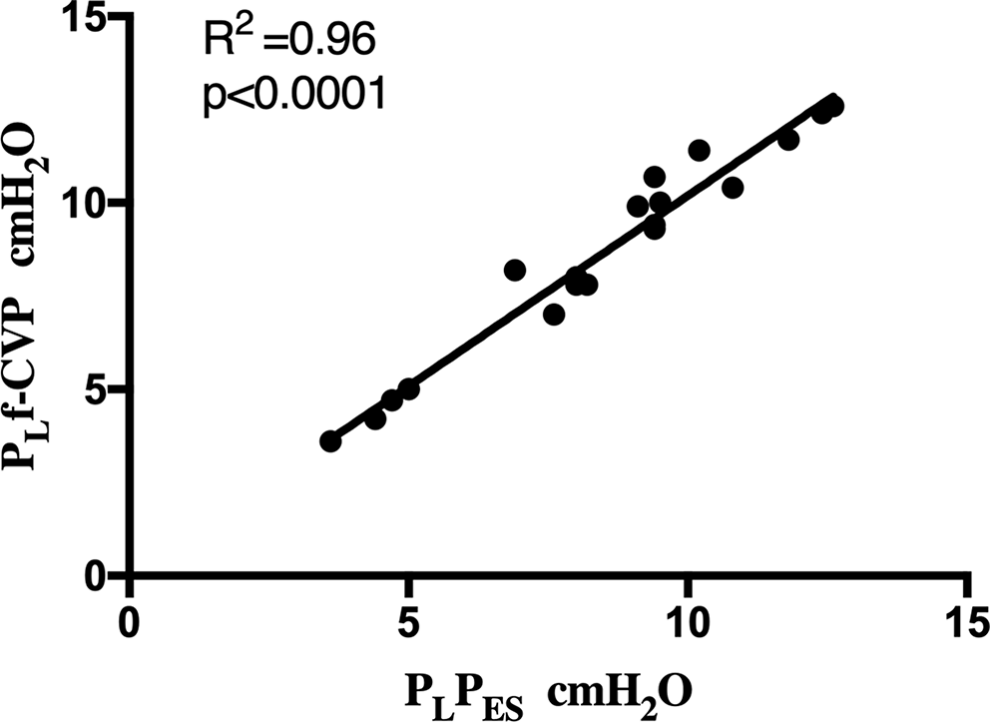
Correlation between P_L_f-CVP and P_ES_. P_L_f-CVP, transpulmonary pressure calculated with filtered central venous pressure; P_ES_, transpulmonary pressure calculated with esophageal pressure. Solid line, linear regression line

Correlations between P_L_CVP and P_L_f-CVP with P_ES_ were *r* = 0.94, *p* < 0.001 and *r* = 0.98, *p* < 0.001 (Fig. 4), respectively. Bland-Altman analysis again revealed a lower bias for P_L_f-CVP than P_L_CVP in comparison to P_L_P_ES_ (0.15, LoA − 0.95, 1.26 cmH_2_O vs. 0.80, LoA − 1.51, 3.12, cmH_2_O respectively).

### Measurements after change in ventilator settings

After P_ES_ evaluation, a variation in the ventilator settings were performed in 16 patients: PEEP was adjusted in four patients, V_T_ in ten and two patients experienced changes in both. In five cases (31%), values of P_L_$\ge$10 cmH_2_O were reduced either by lowering tidal volume < 6 mL/kg predicted body weight (*n* = 4) or by reducing PEEP (*n* = 1). In seven cases (44%), V_T_ was increased to correct respiratory acidosis, keeping P_L_ below 10 cmH_2_O (*n* = 5) or unchanged (*n* = 2). Correlation coefficient between ΔP_ES,_ ΔCVP and Δf-CVP were *r* = 0.49 (*p* = 0.049) and *r* = 0.69 (*p* = 0.003), respectively, and for Bland-Altman analysis mean biases were − 0.67 cmH_2_O (LoA − 2.61, 1.27 cmH_2_O) and − 0.16 cmH_2_O (LoA − 1.46, 1.15 cmH_2_O), respectively. P_L_P_ES_ correlated well with both P_L_CVP and P_L_f-CVP (*r* = 0.93, *p* < 0.001 and *r* = 0.97, *p* < 0.001). Mean bias between P_L_P_ES_ and P_L_CVP was 0.67 cmH_2_O (LoA − 1.27, 2.61 cmH_2_O) and with P_L_f-CVP was 0.16 cmH_2_O (LoA − 1.15, 1.46 cmH_2_O).

**Fig. 5:**
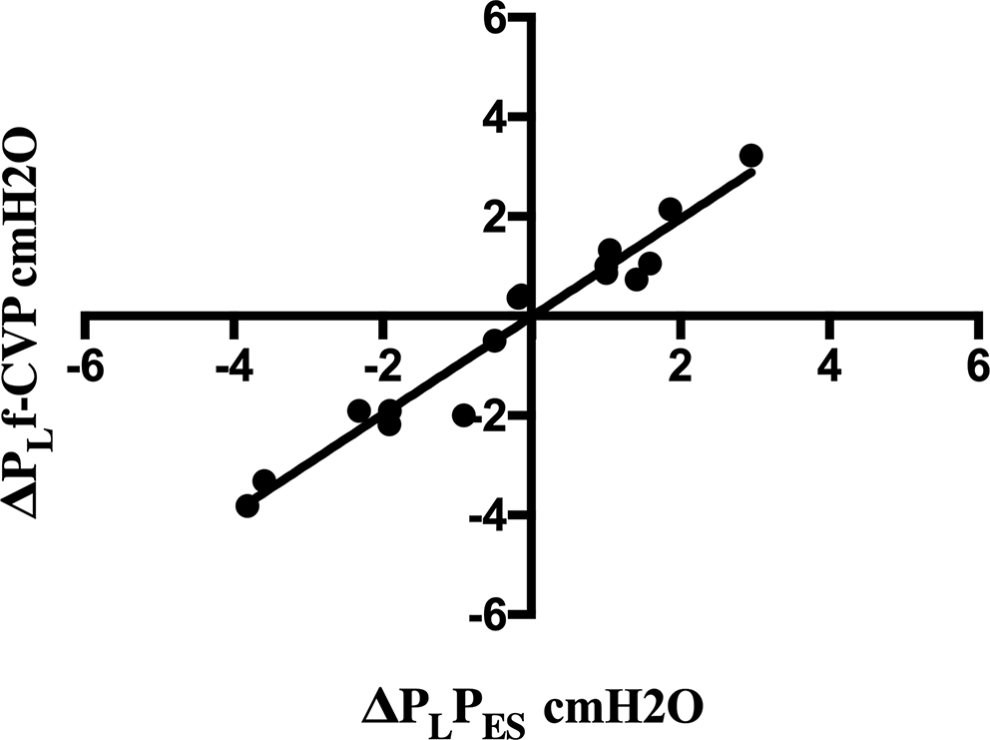
Four-quadrant trend plot of changes of P_L_P_ES_ and P_L_f-CVP from baseline to second measurements. Four-quadrant trend plot representing the relationship between changes (∆) in transpulmonary pressure (P_L_) estimated by esophageal pressure (∆P_L_P_ES_) and filtered central venous pressure (∆P_L_f-CVP). ∆ was calculated by subtracting the first P_L_ (baseline) from the second (after an adjustment in ventilator setting). Twelve ∆P_L_ pairs of measurements were considered because four were excluded from the analysis as at least one ∆P_L_ value was zero. Solid line, line of regression

The change in P_L_ after modification of the ventilator settings (T2-T1) was determined separately for both P_ES_ and f-CVP. As shown in Fig. 5, comparison of the changes measured by the two methods demonstrated a good correlation (*r* = 0.97, *p* < 0.0001). Four P_L_ pairs were excluded from the analysis as at least one P_L_ value was zero, i.e., P_L_ remained unchanged after the adjustment of MV. The concordance of P_L_ was 100% (12 of 12 pairs of P_L_ agreed).

### Diagnostic performance

During baseline measurements, in five patients P_L_P_ES_ was ≥ 10 cmH_2_O; the same patients were identified using the CVP and f-CVP formulas. P_L_f-CVP was ≥ 10 cmH_2_O in other three patients, while P_L_CVP in the other five, instead in the same cases P_L_P_ES_ revealed lower values within normal range. After the change in ventilatory settings that were applied in four patients, in three cases P_L_P_ES_ was reduced below 10 cmH_2_O and in one patient, although lower, remained above this cut-off. In the same patients P_L_f-CVP acted identically (after the changes in ventilatory settings P_L_f-CVP had dropped in all four cases, in three of them below the established cut-off), while P_L_CVP resulted below 10 cmH_2_O in only two of the four cases.

For the baseline measurements, P_L_fCVP discriminated high P_L_ value with an area under the ROC curve of 0.97 (SD, 0.06) (AUC difference = 0.03 [0.03; 0.10], *p* = 0.32); sensitivity was 100%, specificity 80% with positive predictive value of 62.5% and negative predictive value of 100%.

### Low vs. high CVP

Considering all the 36 measurements together, the median value of end-expiratory CVP was 20.5 cmH_2_O. In the low CVP (CVP lower than 20.5 cmH_2_O) group (18 measurements), Δf-CVP maintained a good correlation with ΔP_ES_ (*r* = 0.81, *p* < 0.01), mean bias was 0.16 (LoA − 0.98, 1.29) cmH_2_O, while ΔCVP showed a poor correlation (*r* = 0.30, *p* = 0.27) and a worse accuracy (mean bias 0.8, LoA − 1.33, 2.89 cmH_2_O). In the high (CVP equal or higher than 20.5 cmH_2_O) group (18 measurements) correlation between Δf-CVP and ΔP_ES_ was moderate, although significant (*r* = 0.55, *p* = 0.02), for ΔCVP, instead, became weak (*r* = 0.23, *p* = 0.36). Δf-CVP, again, performed well as accuracy (mean bias 0.16, LoA − 1.10, 1.41 cmH_2_O) compared to ΔCVP (mean bias 0.72, LoA − 1.49, 2.93 cmH_2_O).

## Discussion

In this study, a new approach to estimate respiratory variations of P_ES_ was proposed, using the digital analysis of CVP curves in patients with acute respiratory failure under controlled invasive mechanical ventilation. Δf-CVP showed a good and significant correlation with ΔP_ES_ and could estimate it with accuracy and precision, while “rough”, non-filtered, CVP values exhibited worse results.

When compared with the reference P_L_ calculated from P_ES_, P_L_ derived either by f-CVP and CVP methods showed a high correlation. Then, we demonstrated the capability of the f-CVP method to track variations in P_L_ when ventilator settings were modified. Finally, we were able to detect the same cases of unsafe high P_L_ level as identified with the reference P_ES_ method.

Assessment of P_L_ allows to discriminate lung from chest wall mechanics and consequently titrate mechanical ventilation, although often neglected in clinical practice due to technical and methodological difficulties in using esophageal balloon [1]. Almost all mechanically ventilated patients have a central venous access [29] and we showed that the presence of a correctly placed CVC allowed, together with the presented algorithm of processed CVP, to obtain an estimation of respiratory swings of Ppl and P_L_ without further devices. The idea of using the superior vena cava as an alternative site to the esophagus to record pleural pressure goes back to the 70’s: conflicting results emerged about the correlation between respiratory changes in CVP and P_ES_, and CVP was found to perform better [9], worse [10, 30, 31] or as good as P_ES_ [32] in providing reliable measure of Ppl. Since different catheter-manometer systems were employed and CVP signal was not always processed, we cannot ultimately speculate about these past results. However, some differences between methods employed in the negative studies need to be cited, as a possible explanation of the different results obtained. Ostrander et al. [10] reported that in ten spontaneously breathing dogs ∆Ppl was transmitted to the vena cava not only with attenuation, but also with a significant temporal delay that could alter its accuracy. This phase lag was also seen in thirteen healthy spontaneously breathing subjects [31] and was interpreted as a consequence of the pressure-raising effects of increased inspiratory venous return. This delay was abolished when external airway resistances were added, simulating a respiratory distress, where instead prevailed a fall in intrathoracic pressure during inspiration and also the increased P_ES_ amplitudes were paralleled by similar increases in CVP amplitudes. In our investigation we didn’t observe such a significant delay, probably because we studied paralyzed patients under controlled mechanical ventilation during respiratory pauses, with no airway flow, in which the effect of an eventual phase lag would be not significant. Although physiologically sound and clinically appealing, the idea of using the CVP swings (ΔCVP) as a surrogate of Ppl fluctuations still seems experimental. Recently, this approach has been reevaluated [33]: various authors have reported good and significant correlation between ΔCVP and ΔP_ES_, although a poor agreement, and, in patients breathing spontaneously, ΔCVP was related to the level of inspiratory effort [34, 35]. Very recent experts statement suggested that swings in CVP could be monitored as surrogate of work of breathing to detect strenuous inspiratory efforts and prevent patient-self-induced lung injury (P-SILI) [36]. In our study, thanks to the application of a filtered CVP waveform, the estimation of ΔP_ES_ by ΔCVP was improved in comparison to non-filtered CVP values. A possible explanation for the better results in terms of correlation and accuracy with f-CVP may be related to superimposed cardiac oscillation and to inferences from peaks and troughs upon the CVP waveform during tidal swings that could make accurate reading difficult: these artifacts were cleared with the low-pass filter approach. Instead, transpulmonary driving pressure values were reliable when using both CVP methods (although correlation and accuracy were slightly better with f-CVP). This finding could be easily explained and depend on the calculation of the transpulmonary formula: in our study respiratory swings in Ppl (as measured by P_ES_, CVP and f-CVP) were relatively small and subtracted from a larger common variation in airway pressure.

Another group [12] have recently focused on the same objective proposing an alternative method by applying a corrective factor derived from an occlusion test. Their results were consistent with those of the present study: swings in CVP performed better as a surrogate of ΔP_ES_ when CVP values were “cleared” and the corrected-CVP-derived P_L_ correlated well with P_ES_-derived P_L_. However, the study population significantly differs from ours, as post-cardiac surgery pediatric patients were selected and cases of severe ARDS were not included. Moreover, the calculation of transpulmonary pressure assumed the equivalence between the ratio of ΔPaw to ΔCVP and the ratio of ΔPpl to ΔCVP during an occlusion test, far from being proved in different clinical settings and possible influenced by CVP level at high values. Indeed, due to a blunt transmission of change of Ppl to cardiac cavities and superior vena cava in case of non-compliant heart [37], it has been stated that ΔCVP should not be expected to reflect ΔP_ES_ at higher values of CVP [11]. Thus, we tested our method in cases of high end-expiratory CVP level, based on a threshold derived from the median value found in our study: in cases of CVP above 20.5 cmH_2_O (half of the cases included in the study) the correlation between Δf-CVP and ΔP_ES_ worsened, but remained moderate and significative, and Bland Altman analysis showed a reliable accuracy. This result becomes even more important when considering that a significant group of patients included in the analysis had a diagnosis of severe ARDS (25%), a condition frequently burdened by right heart failure [38], even if in our study we didn’t report its incidence. In contrast to the cited report of Bellamare [11], our findings were consistent with the results of Walling in which ∆CVP and ∆P_ES_ were comparable in cases of higher mean CVP (defined as > 10 cmH_2_O) [9]. Also, ∆CVP remained a reliable index in the identification of high inspiratory efforts without being affected by high mean CVP values [35]. Again, for Lassola et al. the association between ∆CVP and ∆P_ES_ was independent from the value of CVP (with a cut-off for high CVP of 14 cmH_2_O) [34]. One could argue about the different values employed to define a high CVP, but, as reported in a recent expert review, no clear cut-off can be identified [39].

Targeting protective mechanical ventilation also implies the need to stay within a safety P_L_ limit. Transpulmonary driving pressure should be kept below 10–12 cmH_2_O, especially in ARDS patients to minimize the stress applied to an inhomogeneous lung parenchyma [13]. We tested the diagnostic accuracy of P_L_f-CVP in identifying cases of high and potentially unsafe level of P_L,_ as defined by a threshold value of 10 cmH_2_O of P_L_P_ES_: the proposed method worked as a sensitive and specific test to detect patients at risk of VILI with the capacity of rule out such cases.

Superposition of heartbeat-induced pressure changes is commonly seen in the CVP tracing. Hence, we developed a digital filtering able to remove the noise added by the cardiac component on the respiratory waveforms. Similar attempts were made even for P_ES_, because also this waveform is influenced by pressure changes within the pericardium transmitted to the esophageal balloon, hampering the accuracy of the measurements [40–43]. A filtering approach based on fixed cut-off frequencies may suffer from a systematic over-attenuation in amplitude signal and unavoidable information loss because of potential overlap between bandwidths of cardiogenic oscillations and respiratory components. For such reason some authors proposed adaptive filtering techniques [42, 43]. In our study, the drawback related to such bandpass signal did not occur because measurements of CVP were collected during respiratory pauses on MV, in which the bandwidths of heart rate and respiratory component were very distant to each other. Indeed, as one could see in Fig. 1B, the values f-CVP during the respiratory hold maneuvers were slightly larger than the respective during tidal breaths (i.e. the inspiratory pause values of f-CVP were higher than the peak during inspiration and the expiratory pause values of f-CVP were lower than the peak during expiration) in a variable magnitude, but still below 0.5 cmH_2_O in our report. This sort of attenuation depends on the spectral signal overlaps between the respiratory and cardiac component. Such an error could not be neglected if one considers the low-pass filter CVP swings during tidal breaths, because it would imply a significant under-estimation of respiratory efforts in spontaneous breathing patients. So, the feasibility of the proposed technique is restricted to cases where a reliable respiratory hold measure could be obtained. We are aware that during spontaneous breathing this low-pass filter would perform weakly, and a novel adaptive time-variant filtering technique of CVP is being developed [20]. In our study similar digital processing was not attempted for P_ES_ curves because our aim was to propose a method of P_L_ calculation that could do without an esophageal balloon, not to ameliorate the reference one. We are aware that f-CVP estimation of P_L_ was obtained with an off-line analysis which could limit its clinical bedside application. Nonetheless, it is far less time consuming than the reference method, without any need for catheter calibration and could be easily implemented in a real-time monitoring.

Our study has some limitations. First, it is single-center and was conducted in a small population, although it is consistent with a preliminary clinical report of an experimental technique. Second, our method was applied in patients on controlled invasive MV and not tested in patients in spontaneous breathing: future research may involve such category of patients. Third, we measured CVP and P_ES_ in a semi-recumbent position, while in most studies they were measured in supine position [9, 12, 16]. Nevertheless, our analysis was non-interventional and performed in a population of intubated patients with severe respiratory disease, who, as routine standard of care, were maintained in a semi-recumbent position. Other recent studies found a positive correlation in tidal swings of CVP and P_ES_ in semi-recumbent position in a real-life scenario [34, 35]. This approach is supported in literature: Flemale demonstrated that both CVP and P_ES_ swings provide accurate measurements of the change in pleural pressure in different body positions, including supine and seated, if these methods were validated by an occlusion test [32]. In addition, recently, Repessé suggested the semirecumbent over the supine position in the evaluation of esophageal pressure because in the latter P_ES_ is more altered by cardiac artifacts and also tends to overestimate the value of P_ES_ [44].

One could wonder about the feasibility of the proposed technique: indeed, four patients out of twenty-four were excluded. Actually, one patient was erroneously enrolled because of his cardiac arrhythmia, a condition that might interfere with the digital filtering analysis resulting in a disturbed waveform. The other three patients were excluded due to esophageal pressure waveforms impossible to interpret because of superimposed artifacts, while in the same patients the f-CVP signals were readable. This proved again the difficulties of having a readable P_ES_ curve and underlined the need of an alternative method of transpulmonary pressure estimate.

## Conclusions

Respiratory swings in CVP values obtained with a dedicated digital filter could represent a surrogate of ΔP_ES_ and P_L_ calculated with f-CVP was strongly comparable with the reference P_L−_derived esophageal balloon technique. Moreover, the f-CVP method identified patients with high P_L_ levels potentially at risk of VILI. Currently, the proposed technique is an off-line analysis, but could be easily integrated in a bedside monitor. Finally, the reliability of the f-CVP method must be assessed in spontaneous breathing patients.

## Data Availability

The datasets analyzed during the current study are available from the corresponding author on reasonable request.
